# Retraction: Magnetic resonance imaging and ^18^F-fludeoxyglucose positron emission tomography/computed tomography findings of retroperitoneal clear cell carcinoma with an unknown primary site: a case report

**DOI:** 10.3389/fmed.2023.1292298

**Published:** 2023-09-13

**Authors:** Frontiers Editorial Office

The journal retracts the 2022 article cited above.

Following concerns regarding the originality of the discussion in the article, an investigation was conducted in accordance with Frontiers' policies. It was found that the complaint was valid and that the article should be retracted, because of an unacceptable high level of similarity to the discussion section of a different case study (article published by Yang et al., 2022: *Chin J Nucl Med Mol Imaging*, 2022, 42(3):174–176. doi: 10.3760/cma.j.cn321828-20220206-00031).

This retraction was approved by the Chief Editors of Frontiers in Medicine and the Chief Executive Editor of Frontiers. The authors agree to this retraction.

